# A real-world pharmacovigilance analysis of eslicarbazepine acetate using the FDA adverse events reporting system (FAERS) database from 2013 (Q4) to 2024 (Q1)

**DOI:** 10.3389/fphar.2024.1463560

**Published:** 2024-09-20

**Authors:** Huafei Tang, Jing Xu, Xian Zhang, Chunliang Chen, Ge Song, Rui Ma, Jinjing Zhao, Qiang Zhao

**Affiliations:** ^1^ Department of Pharmacy, The 305 Hospital of PLA, Beijing, China; ^2^ Department of Pharmacy, Daping Hospital, Army Medical University, Chongqing, China; ^3^ Department of Neurology, The 305 Hospital of PLA, Beijing, China

**Keywords:** eslicarbazepine acetate, ESL, FDA, pharmacovigilance analysis, AES

## Abstract

**Background:**

The approval of eslicarbazepine acetate (ESL) by the Food and Drug Administration (FDA) in 2013 marked an advancement in the treatment of adult patients with partial-onset seizures. However, there still remains a paucity of real-world studies regarding the adverse events (AEs) associated with this compound. The principal aim of the present study was to scrutinize ESL-related AEs by leveraging data from the US Food and Drug Administration Adverse Event Reporting System (FAERS) database.

**Methods:**

By extracting all available data since the FDA approval of ESL (2013Q4-2024Q1), disproportionality analysis was performed using reporting odds ratio (ROR), proportional reporting ratio (PRR), Bayesian confidence propagation neural network (BCPNN) and multi-item gamma Poisson shrinker (MGPS) algorithms. AE signals that simultaneously met the requirements of all four algorithms were identified as significant positive signals. Demographic information, time of onset and gender-specific signal detection were also examined. In addition, a special screening process for designated medical events (DME) was implemented to focus on the evaluation and comparison of safety signals within DME and System Organ Classification (SOC) level, as well as SMQ (Standardised MedDRA Queries) level. Stratified analysis by logistic regression is employed to examine the variations across different gender (male and female) and age groups (<18 years old, 18–64 years old, >65 years old).

**Results:**

A total of 5,719 AE reports and 1,907 reported cases were obtained. ESL related AEs were identified in relation to 27 SOCs, among which the significant positive SOCs were nervous system disorders, injury poisoning and procedural complications, etc. There were 86 severely disproportional preferred terms that complied with the four algorithms. Most AEs occurred within the first month after treatment. According to the 86 valuable positive signals with DME screening results, 3 signals of dermatitis exfoliative, stevens-johnson syndrome, drug reaction with eosinophilia and systemic symptoms were consistent with PT signals on the DME-list, with the 3 PTs focusing on skin and subcutaneous tissue disorders and hypersensitivity. Males are more commonly affected by seizures than females. Seizures, hyponatremia, and confusional states were more frequently observed in the elderly population, while aggression, irritability, DRESS (drug reaction with eosinophilia and systemic symptoms), and abnormal behavior were found to be more common in the pediatric population. Both the children and elderly groups exhibited a higher proportion of agitation than the adult group.

**Conclusion:**

Our research enhances the safety and tolerability profile of ESL, but the clinical use of ESL should be noticed and avoided in relation to AEs since it raises the risk of dermatitis exfoliative, stevens-johnson syndrome. Particular attention should be paid to DRESS in children and hyponatremia in the elderly.

## Introduction

Epilepsy is one of the oldest known diseases, with its earliest documented records dating back to 4000 BCE (Kaculini et al., 2021). Despite the recognition of its mechanism and the development of treatments, it remains a common chronic disease of the nervous system that presents significant challenges to the health of patients. The latest data from the Global Disease Burden (GBD) 2021 show that epilepsy has a globally prevalence of 308.9 (95%UI 236.2-390.1), deaths of 1.7 (95%UI 1.5-1.9) and disability-adjusted life years (DALYs) of 183.9 (95%CI 141.0-237.2) in age-standardized rates (per 100,000 people) (GBD 2021 Nervous System Disorders Collaborators, 2024). The focus and complexity of epilepsy lies in the diagnosis and classification of the disease, which is necessary to guide the best possible management of the condition (GBD 2021 Nervous System Disorders Collaborators, 2024; Thijs et al., 2019). In terms of treatments, options encompass the utilization of the ketogenic diet, pharmaceutical intervention, and surgical procedures, with anti-epileptic drugs (AEDs) being the most important method. Unfortunately, epilepsy is not yet curable, but many people can achieve seizure-free with the appropriate treatment. Nevertheless, approximately one-third of patients continue to experience seizures despite the administration of dual AEDs or in the presence of intolerable side effects, a condition commonly referred to as drug-resistant epilepsy (Asadi-Pooya et al., 2023). Hence, a compelling need for the continuous innovation and progression of pioneering AEDs still exists.

Eslicarbazepine acetate (ESL), a voltage-gated sodium channel antagonist, was initially approved by the European Medicines Agency (EMA) in 2009 and subsequently by the US Food and Drug Administration (FDA) in 2013 for the adjunctive treatment of patients with partial onset seizures. Being the third generation of the dibenzazepine carboxamide family, this compound possesses structural advantages with a hydroxy group rather than a keto group in the 10th position of the ring, which leads to a safer profile by producing less toxic metabolites and minimizing enzymatic induction of the cytochrome P450 (CYP) system (Galiana et al., 2017). The existing pre- and post-clinical trials indicate that ESL has comparable efficacy to carbamazepine and oxcarbazepine, with the additional benefits of improved tolerability and patient compliance (Lattanzi et al., 2018). However, there is a paucity of evidence from large population research on the adverse events (AEs) of ESL, particularly in relation to children, older individuals over the age of 65, and individuals of different genders. Consequently, the objective of this study is to analyse adverse event signals associated with ESL using the FDA Adverse Event Reporting System (FAERS) in order to provide insights for its clinical application from a real-world perspective.

## Materials and methods

### Data source

**FIGURE 1:**
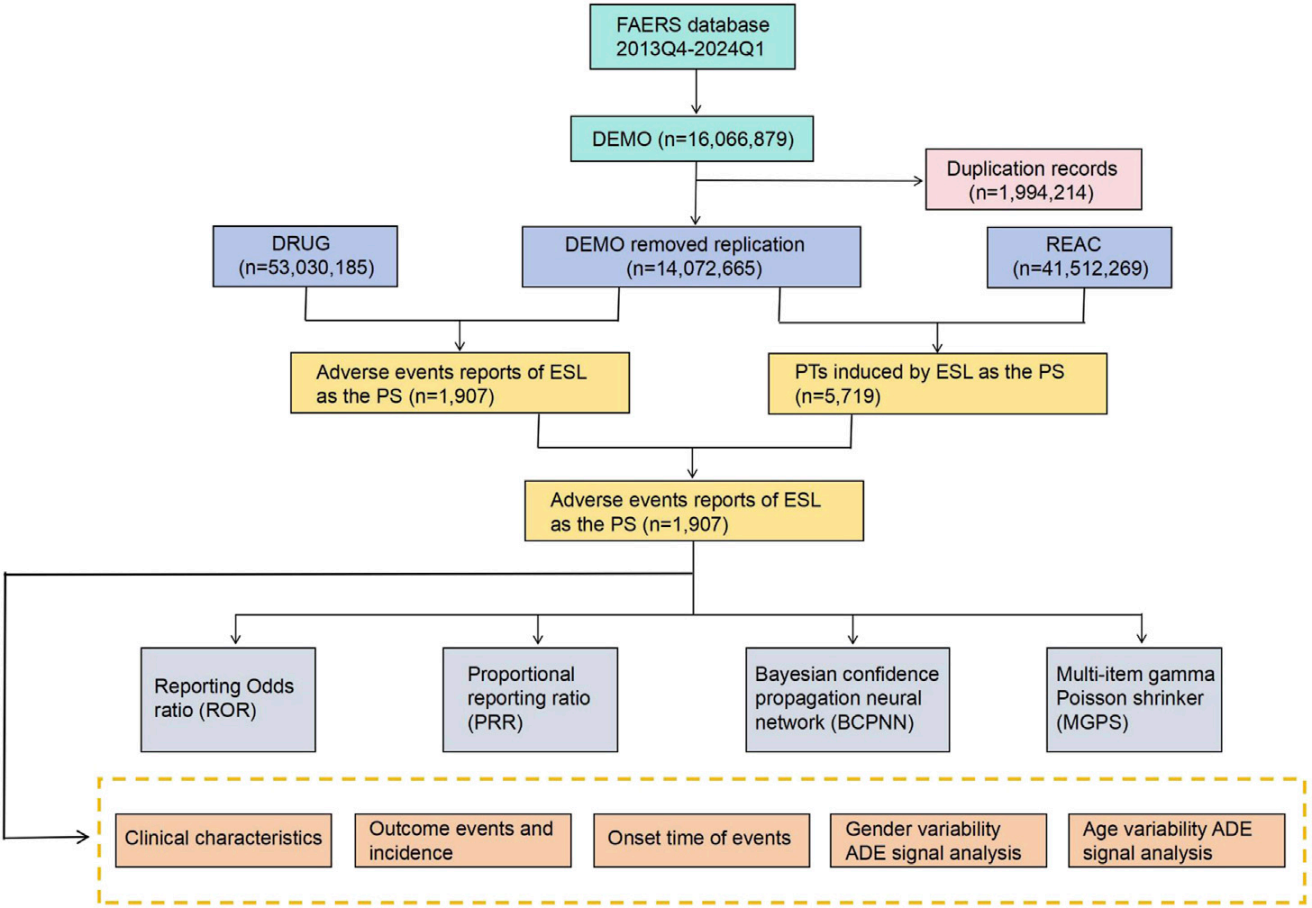
The process of searching and analyzing eslicarbazepine acetate-associated adverse events from the US Food and Drug Administration (FDA) Adverse Event Reporting System (FAERS), ADE: adverse drug events.

The FAERS database is the largest publicly accessible pharmacovigilance database in the world, receiving AE reports from a multitude of sources across the globe. Its extensive size and global coverage make it particularly well-suited for identifying potential associations between drugs and AEs. All the data for this retrospective pharmacovigilance study on ESL was retrieved from the FAERS between the fourth quarter (Q4) of 2013 and the first quarter of 2024 (Q1). The steps for data processing include the followings: 1) To eliminate duplicates prior to statistical analysis, the higher PRIMARYID was selected when the CASEID and FDA_DT were identical, and the most recent FDA_DT was chosen when the CASEID and FDA_DT were identical, following by removed the deleted cases; 2) To guarantee that all reports of ESL have been extracted from the Drug file, we employ the Medical Subject Headings (MeSH) to search for all ESL referent names, including trade names such as Zebinix, Aptiom, Zebinix eslicarbazepine acetate, compound codes and chemical formulas; 3) To enhance the credibility of the results, we only extract reports in which ESL was deemed to cause adverse events with a role_cod of PS (Primary Suspect); 4) Using the Medical Dictionary for Regulatory Activities (MedDRA) (version 26.1) to identify each individual report of ESL AEs at the Preferred Term (PT), System Organ Class (SOC) and Standardised MedDRA Queries (SMQ) levels; 5) The inaccurate and missing records of date were eliminated, and the following formula was used to calculate the time-to-onset of AEs brought on by ESL: (Time-to-onset = Adverse event onset date - start date of ESL use). The methodology employed for the identification and analysis of ESL-associated AEs from the FAERS database is illustrated in Figure 1.

### Data analysis

Our study employed disproportionality analysis to detect AEs as signals. This approach assesses the relative occurrence of AEs associated with a specific medication in comparison to all other pharmaceuticals. Four methods were employed for AE signal mining, including the Reporting Odds Ratio (ROR) method, the Medicines Healthcare Products Regulatory Agency (MHRA) method, the Bayesian Confidence Propagation Neural Network (BCPNN) method, and the Multi-Item Gamma Poisson Shrinker (MGPS) method (see in Table 1). A significant signal for PTs is detected when the specific AE occurrence rate of the target medication exceeds the background frequency and simultaneously meets the threshold or criteria of the four indices mentioned above (see in Table 2). To evaluate some serious and specific safety events related to ESL administration, this study further screened the Designated Medical Events (DME) list for valuable positive signals. Reporting odds ratio and stratified analysis by logistic regression is employed to examine different gender (male and female, see in Table 3) and age groups (<18 years old, 18–64 years old, >65 years old) respectively, in order to determine if there are variations in the occurrence of AEs. All analyses were performed using R software version 4.3.2 (R Foundation for Statistical Computing, Vienna, Austria). p < 0.05 was considered statistically significant, and the Bonferroni correction method was used for multiple comparisons.

**TABLE 1:** Detailed formulas for disproportionality analysis.

Methods	Formula	Signal standard
ROR	ROR = ad/bc ROR 95%CI = eln (ROR)±1.96 (1/a+1/b+1/c+1/d)^0.5^	a≥3 lower limit of ROR 95%CI > 1
MHRA	PRR = a (c + d)/c (a+b) PRR 95%CI = eln (PRR)±1.96 (1/a-1/(a+b)+1/c-1/(c + d))^0.5^	a≥3 lower limit of PRR 95%CI > 1
BCPNN	IC = log_2_ [a (a+b + c + d)]/[(a+b) (a+c)] γ = γ_ij_ [(N+α) (N+β)]/[(a+b+α_i_) (a+c+β_j_)] E (IC) = log_2_ [(a+γ_ij_) (N+α) (N+β)]/[(N+γ) (a+b+α_i_) (a+c+β_j_)] V(IC)=(log2)^−2^{(N-a+γ-γ_ij_)/[(a+γ_ij_) (1 + N+γ)] + (N-a-b+α-α_i_)/[(a+b+α_i_) (1 + N+α)] + (N-a-c+β-β_i_)/[(a+b+β_i_) (1 + N+β)]} SD = 2(V(IC))^0.5^ IC-2SD = E (IC)-2SD	a≥3 IC-2SD > 0
MGPS	EBGM = a (a+b + c + d)/[(a+c) (b + d)] EBGM05 = eln (EBGM)±1.96 (1/a+1/b+1/c+1/d)^0.5^	a>0 EBGM05 > 2

Note: γ, γ_ij_ are the Dicichlet distribution parameter; α_i_, α, β_j_, β are Beta distribution parameter; SD, is the standard deviation; IC-2SD, is the lower limit of IC, 95% CI; hypothesis α = β = 2, γ_ij_ = β_j_ = α_i_ = 1; ROR: reporting odds ratio; MHRA:medicines healthcare products regulatory agency; PRR: proportional reporting ratio; BCPNN: bayesian confidence propagation neural network; MGPS: Multi-Item Gamma Poisson Shrinker; EBGM: empirical bayesian geometric mean; EBGM05: lower limit of EBGM, 95%CI., 95% CI: 95% Confidence Interval.

**TABLE 2:** Two-by-two contingency table for disproportionality analysis.

	Number of target adverse events reports	Number of other adverse events reports	Total
ESL	a	b	a+b
Other drugs	c	d	c + d
Total	a+c	b + d	N = a+b + c + d

**TABLE 3:** Two-by-two contingency table for disproportionality analysis (stratified by case sex).

	Number of target adverse events reports	Number of other adverse events reports	Total
Male	a	b	a+b
Female	c	d	c + d
Total	a+c	b + d	N = a+b + c + d

## Results

### General characteristics

The FAERS database yielded a total of 14, 072, 665 reports between 2013 (Q4) and 2024 (Q1) after the removal of duplicates. We extracted 1,907 AEs reports related to ESL as PS, with 5,719 AE records. The general descriptions were presented in Table 4. It appears that ESL-related AEs were more common in females than males, with the exception of those whose gender is unknown. No discernible correlation was identified between body weight level and the occurrence of AEs. Among individuals with known ages, AEs were concentrated in the age group of 18–64.9 years (24.6%), followed by those aged 65–85 years (5.3%). The AE reports were primarily self-sponsored by patients (78.3%), in a manner similar to other AEDs that necessitate long-term medication. For severe outcomes, the most frequently reported were OT (Other serious medical events, 51.8%) and HO (Hospitalization, 13.8%). With regard to the report country, America (78.8%) submitted the greatest number of reports, followed by Portugal (8.9%), France (3.3%), Spain (2.4%), and Canada (2.0%). During the study period, the number of reports of ESL-related AEs exhibited a gradual increase from 2014, reaching a peak in the year 2019, followed by a subsequent decline to 2023.

**TABLE 4:** Clinical characteristics of eslicarbazepine acetate associated reports from the FAERS database (2013 Q4 to 2024 Q1).

Characteristics	Case Number, n	Proportion, %
Number of events	1,907	
Sex		
Female	799	41.6
Male	545	28.6
Unknown	563	29.5
Weight (kg)		
<50	25	1.3
>100	48	2.5
50–100	174	9.1
Unknown	1,660	87.0
Age (years)		
<18	75	3.9
18–64.9	470	24.6
65–85	101	5.3
>85	12	0.6
Unknown	1,249	65.5
Reporter		
Consumer (CN)	1,493	78.3
Health professional (HP)	56	2.9
Physician (MD)	220	11.5
Other health professional (OT)	98	5.1
Pharmacist (PH)	26	1.4
Unknown	14	0.7
Serious Outcome		
Death (DE)	56	2.6
Hospitalization (HO)	302	13.8
Disability (DS)	21	1.0
Congenital anomaly (CA)	10	0.5
Life-threatening (LT)	37	1.7
Required intervention to prevent permanent impairment/damage (RI)	1	0.0
Other serious medical events (OT)	1,136	51.8
Unknown	632	28.8
Reported countries (Top 5)		
America (US)	1,503	78.8
Portuguese (PT)	169	8.9
France (FR)	62	3.3
Spain (ES)	46	2.4
Canada (CA)	39	2.0
Reporting year		
2013Q4	8	0.42
2014	38	1.99
2015	119	6.24
2016	129	6.76
2017	206	10.80
2018	287	15.05
2019	298	15.63
2020	221	11.59
2021	255	13.37
2022	128	6.71
2023	144	7.55
2024Q1	74	3.88

### Risk signal detection results

**FIGURE 2:**
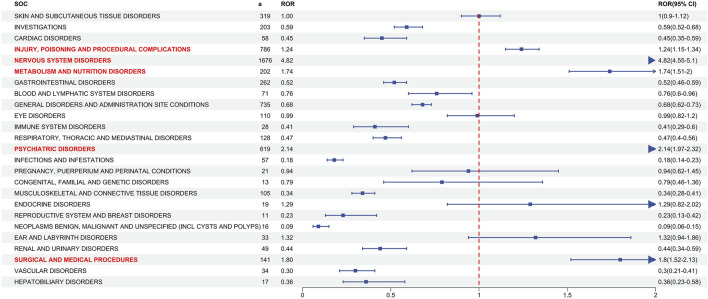
Signal detection at the SOC level. The ROR values and their 95% confidence intervals (95% CI) are visualized. The SOCs exhibiting positive signal values were highlighted in red for clarity. The blue arrows signify that the lower limit of the 95% confidence interval of the ROR exceeds 2. a: number of the cases, SOC: System Organ Class, ROR, reporting odds ratio. The p-value is adjusted with Bonferroni correction method.

Figure 2 depicts signal strengths and reports of ESL at the SOC level, with 27 SOCs affected by ESL-induced AEs. The significant SOCs for which at least one of the four algorithms met the criteria were injury, poisoning, and procedural complications (SOC: 10,022,117), nervous system disorders (SOC: 10,029,205), metabolism and nutrition disorders (SOC: 10,027,433), surgical and medical procedures (SOC: 10,042,613), and psychiatric disorders (SOC: 10,037,175).

**FIGURE 3:**
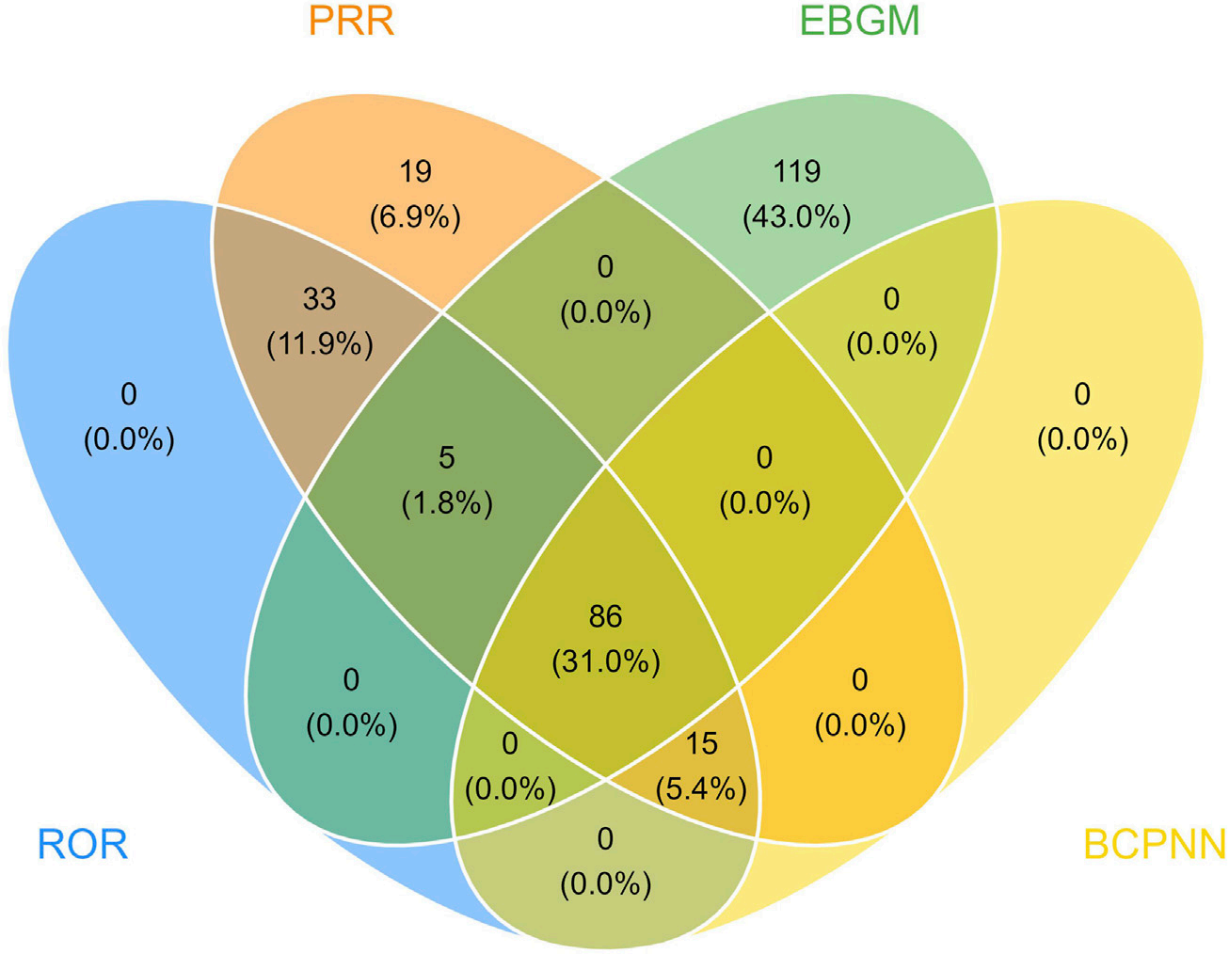
The meticulous application of four distinct methodologies resulted in the identification of 86 efficacious PTs. Out of an assemblage of 1,907 signals, the ROR method surfaced 139 relevant signals, the PRR method identified 158, the BCPNN method segregated 101, and the EBGM method segregated 210 effective signals.

**FIGURE 4:**
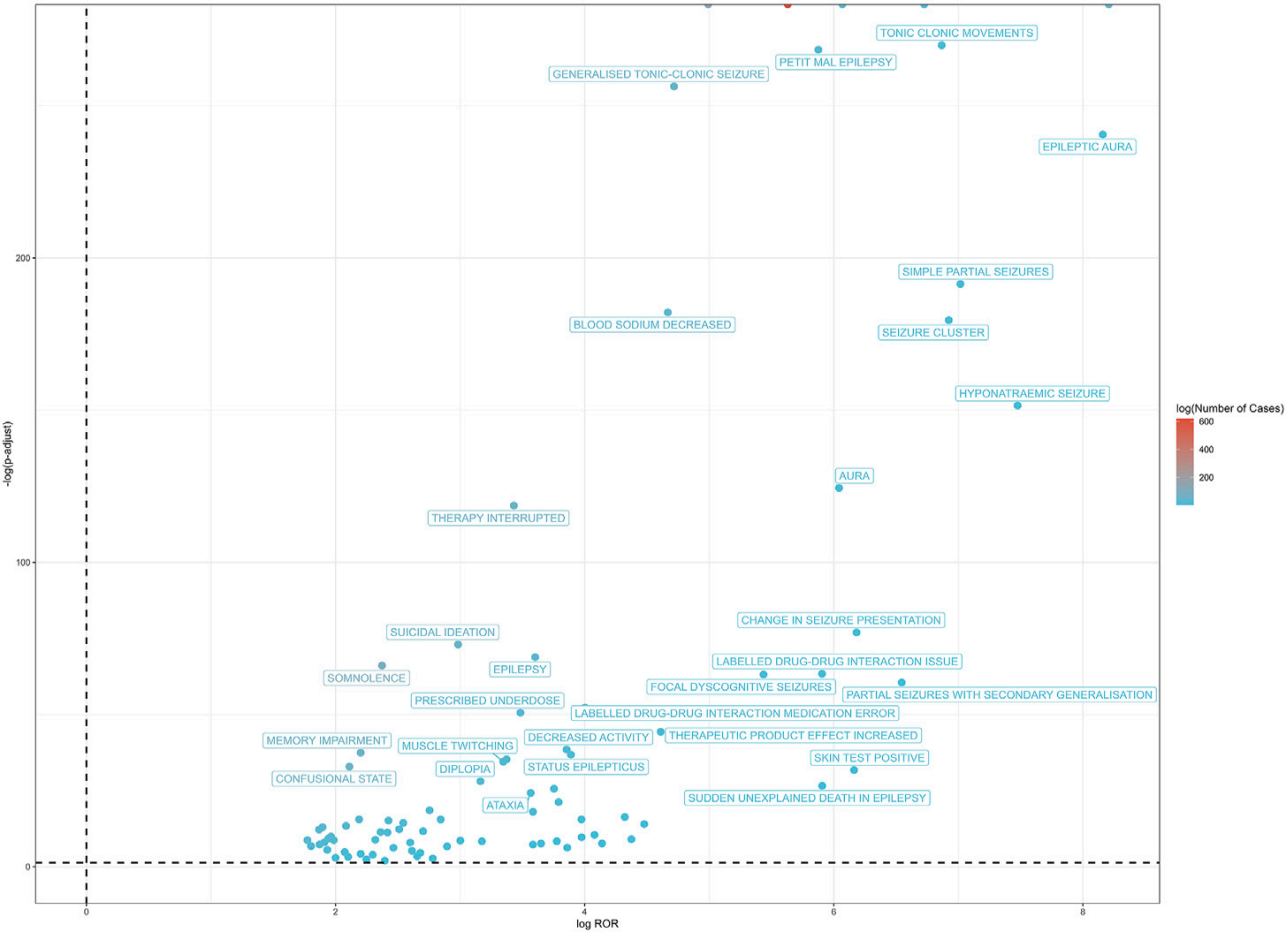
The positive risk signal volcano plot for ESL. The horizontal coordinate shows the log2 ROR value and the vertical coordinate indicates the adjusted p-value after -log10 conversion. Significant signals are highlighted and annotated in prominent colors. Five of the PTs displayed values above the upper limit of the figure, including hyponatremia, seizure, partial seizures, brain operation, and drug dose titration not performed from left to right. The p-value is adjusted with Bonferroni correction method.

**FIGURE 5:**
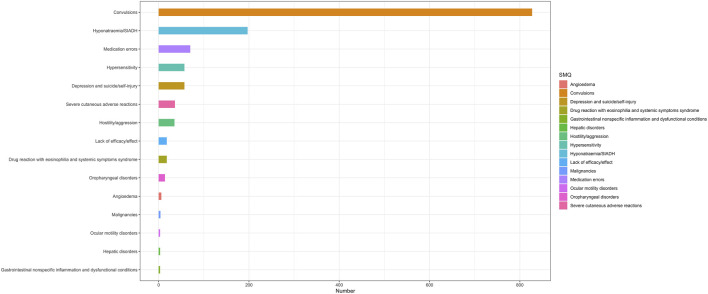
The SMQ attribution of PTs that simultaneously satisfy the 4 methods of disproportionality analysis with positive signal values. 34 of the 86 positive PTs were matched with Narrow SMQ and classified into the corresponding SMQ. SMQ: Standardised MedDRA Queries, MedDRA: Medical Dictionary for Regulatory Activities.

At the PT level, in accordance with the specifications of four distinct algorithms, they were identified as ROR (139 positive PTs), PRR (158 positive PTs), BCPNN (101 positive PTs) and EBGM (210 positive PTs), resulting in a total of 86 effective PTs (simultaneously met four algorithms criteria, 31.0% for all positive PTs) (Figure 3; Supplementary Table S1). A volcano plot was generated for the 86 effective PTs, displaying the log2-transformed ROR values on the horizontal axis and the log10-transformed corrected p-values (P.adj, adjusted by Bonferroni) on the vertical axis (Figure 4). The ROR indicated the strength of the association between ESL and AEs, with PTs on the right side (higher log2-transformed ROR) exhibiting a stronger relationship than those on the left. Five of the PTs displayed values above the upper limit of the figure, which was attributed to the presence of small p-values. The five PTs were as follows: hyponatremia (PT: 10,021,036), seizure (PT: 10,039,906), partial seizures (PT: 10,061,334), brain operation (PT: 1,0,061,732), and drug dose titration not performed (PT: 10,074,906) from left to right. It was notable that certain PTs could be grouped together based on their similar presentation or common pathological pathway. Subsequently, the SMQ level was employed to categorize the PTs (34 PTs with Narrow SMQ match), with the creation of a distribution map serving to illustrate this categorization in greater detail (Figure 5; Supplementary Table S2). The top five SMQs were convulsions (SMQ: 20,000,079, 15 PTs, n = 827), hyponatremia/SIADH (SMQ: 20,000,141, 3 PTs, n = 197), medication errors (SMQ: 20,000,224, 2 PTs, n = 70), hypersensitivity (SMQ: 20,000,214, 7 PTs, n = 57), and depression and suicide/self-injury (SMQ: 20,000,035, 1 PT, n = 57).

### DME list screening

3 of the 86 positive signals, including dermatitis exfoliative, stevens-johnson syndrome, and drug reaction with eosinophilia and systemic symptoms, were matched with the DME list. All of these signals focus on skin and subcutaneous tissue disorders (SOC level) and hypersensitivity (Narrow SMQ level). The results indicated the presence of five positive signals in AEs related to skin and subcutaneous tissue disorders (Table 5). The highest signal value was observed in dermatitis exfoliative, with a ROR value of 13.7 and a lower 95% CI of 5.14.

**TABLE 5:** DME list screening results.

PT	a	ROR	Lower limit of 95% CI	Instructions included or not
Dermatitis exfoliative	4	13.7	5.14	Yes
Toxic skin eruption	6	7.44	3.34	Yes
Drug reaction with eosinophilia and systemic symptoms	18	6.75	4.25	Yes
Rash maculo-papular	12	6.51	3.69	Yes
Stevens-johnson syndrome	8	5.52	2.76	Yes

PT: preferred term, a: number of the PT, reports, ROR: reporting odds ratio, DME: designated medical events, 95% CI: 95% confidence interval. The bold PTs, are positive and matched with the DME, list.

### Subgroup analysis

#### Gender-differentiated signal detection

**FIGURE 6:**
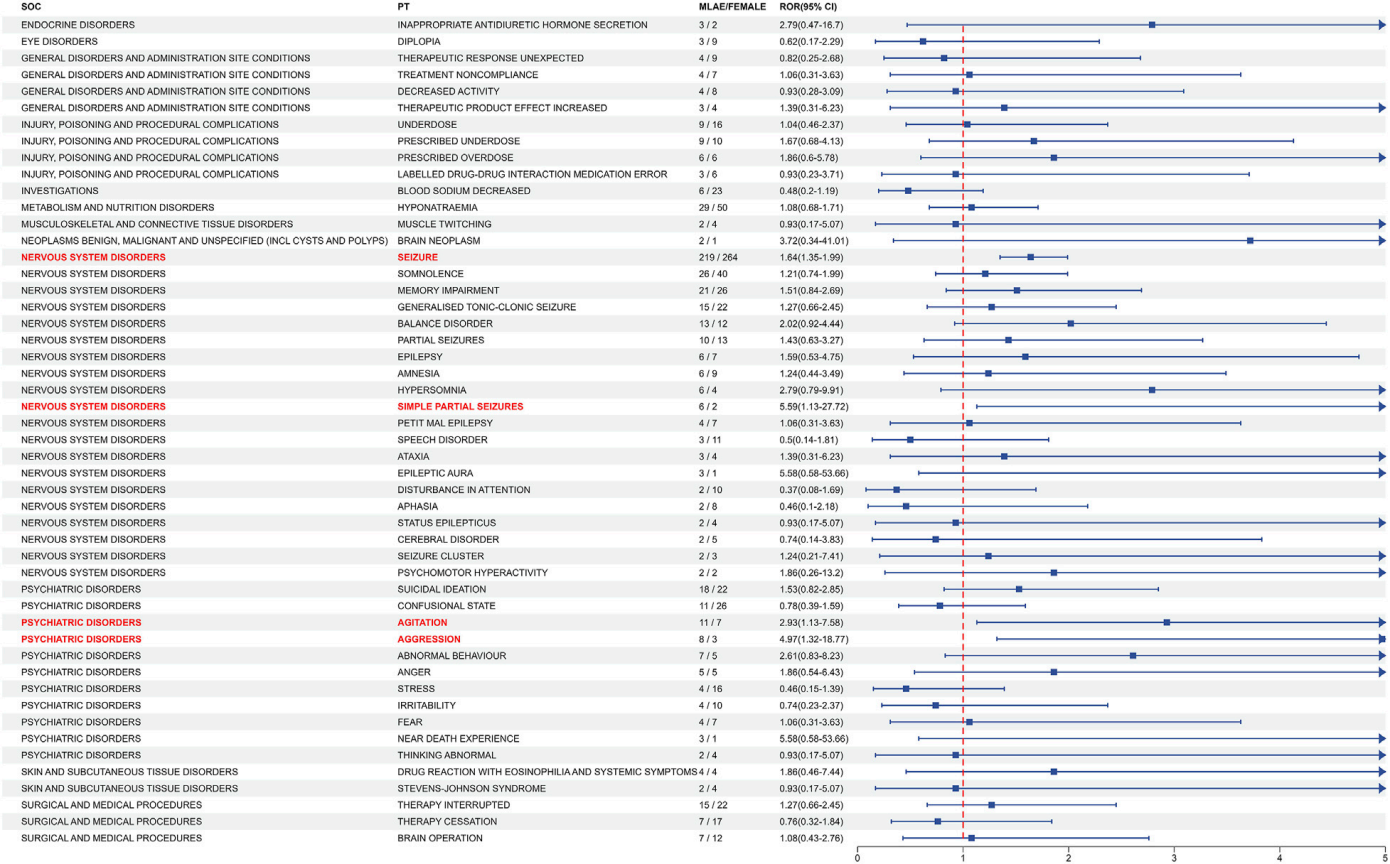
Analysis of gender-differentiated risk signals in ESL. The top 50 PTs in case number were displayed in order of SOC alphabet, with positive gender-related ADEs highlighted in red. ROR: Reporting odds ratios, 95% CI: 95% confidence interval. The blue arrows signify that the lower limit of the 95% confidence interval of the ROR exceeds 5. The p-value is adjusted with Bonferroni correction method.

In order to ascertain whether gender is a factor in the AEs of ESL, the ROR method was employed to identify the 86 PTs with a disproportionate AE incidence between males and females. Figure 6 presents the initial 50 PTs in order of incidence number, categorized by SOC. A total of 4 gender-differentiated signals for males involving 2 SOCs were generated by gender-differentiated analysis. In the context of nervous system disorders, males were more commonly affected by seizure (PT: 10,039,906, ROR = 1.64, 95% CI: 1.35–1.99) and simple partial seizures (PT: 10,040,703, ROR = 5.59, 95% CI: 1.13-27.72). For psychiatric disorders, high-risk ADEs including agitation (PT: 10,001,497, ROR = 2.93, 95% CI: 1.13–7.58) and aggression (PT: 10,001,488, ROR = 4.97, 95% CI: 1.32–18.77) were more common in males. No significant differences were identified between the various genders with regard to the other PTs.

#### Age subgroup analysis

**FIGURE 7:**
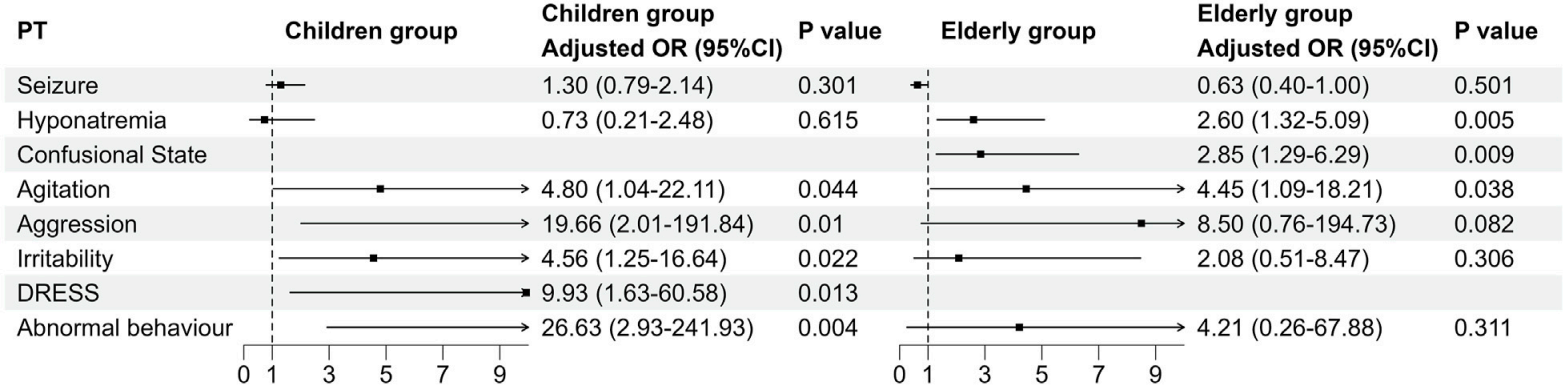
Logistic regression of the different age groups for the 86 positive PTs. The adult group was the reference group. The OR was adjusted for age and gender. Eight of the 86 PTs were statistically significant, as illustrated in the figure. The adjusted OR values for the “confusional state” of the children group and the “DRESS” of the elderly group are unavailable due to the limited number of positive cases. PT, Preferred term; OR, Odds ratio; DRESS, Drug reaction with eosinophilia and systemic symptoms.

To investigate the relationship between age and AEs of ESL, we stratified age into three subgroups: children (aged <18 years), adult (aged 18–65 years), and elderly (aged >65 years). The adult group was considered to be the reference group. A total of 650 reports with complete and relevant information were extracted from the 1,907 AE reports, including 256 males, 394 females, 73 children, 456 adults and 112 elderly. Logistic univariate and multivariate regression (considering age and gender factors) analyses were then conducted on the 86 positive PTs, respectively (see in Figure 7; Supplementary Table S3). It is important to note that when the number of occurrences within a specific subgroup is insufficient, it is not possible to calculate the odds ratio (OR) value. The results demonstrated that the occurrence of seizure (PT: 10,039,906), hyponatremia (PT: 10,021,036) and confusional state (PT: 10,010,305) was more prevalent in the elderly group than in the adult group. Notably, seizure was significant with crude OR (0.64, 95%CI 0.40–0.99, p = 0.049) for the elderly group. Conversely, aggression (PT: 10,001,488), irritability (PT: 10,022,998), drug reactions with eosinophilia and systemic symptoms (PT:10,073,508), and abnormal behaviour (PT: 10,061,422) were more frequently observed in the children group than in the adult group. Notably, agitation (PT: 10,001,497) was more common in both the children group and the elderly group than in the adult group.

#### Onset time of events

**FIGURE 8:**
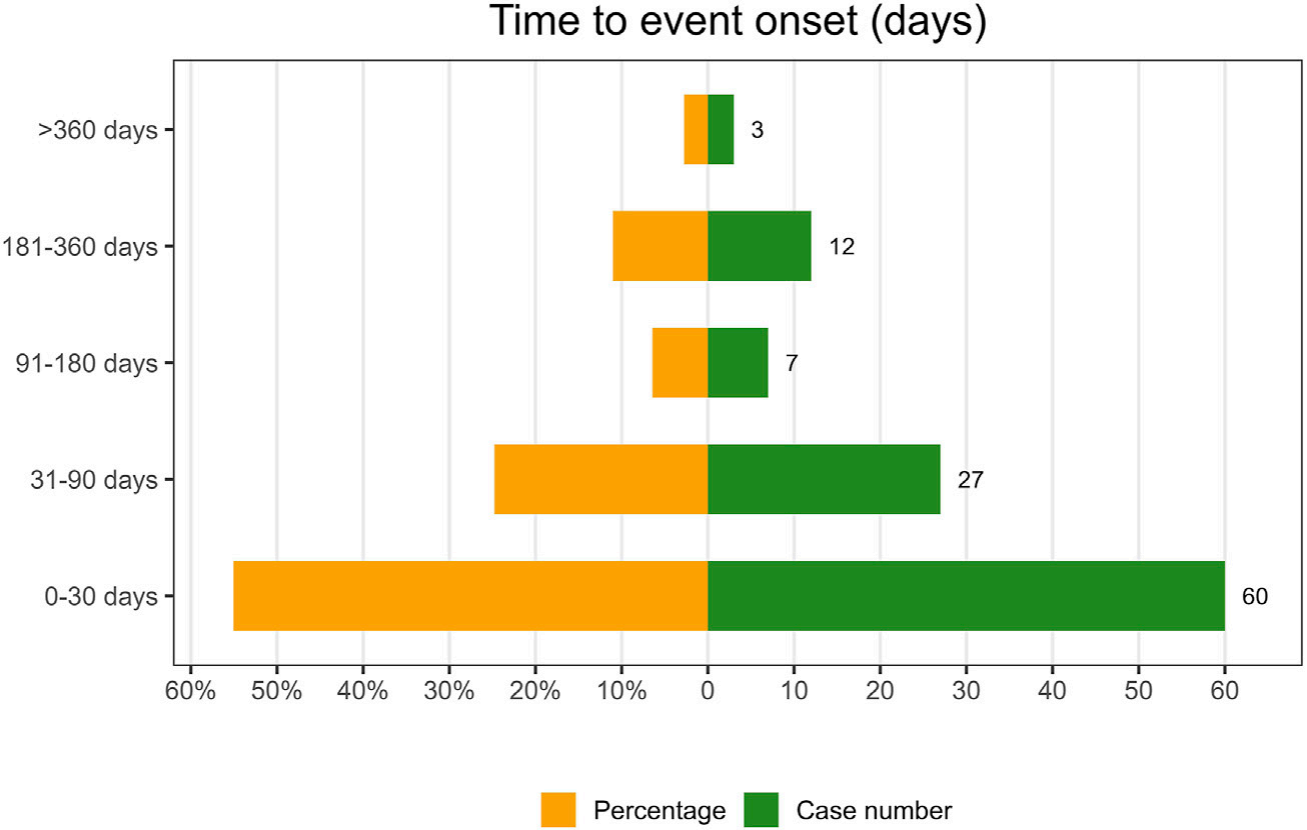
Time to onset of ESL-related AEs.

Following the exclusion of inaccurate, missing, or unknown reports of onset, a total of 109 AEs were collected. The median TTO (Time to onset) was determined to be 27 days (interquartile range [IQR] 8–62 days). As illustrated in Figure 8, the majority of cases occurred within the initial month (n = 60, 55.05%) of ESL administration. The number of AEs decreased over time, with 27 AEs (24.77%) occurring in the 31–90 days and 7 AEs (6.42%) in the 91–180 days. Notably, in 2.75% of cases, AEs could still occur even after 1 year of treatment with ESL. To ascertain whether the risk of ESL-associated AEs exhibited a temporal trend, we conducted Weibull distribution tests. In the context of the overall analysis, the calculated shape parameter (β) was 0.69, with the upper limit of its 95% confidence interval (CI) being 0.78. Both values were below 1, indicating a decline in the prevalence of AEs over time (Early failure type, see in Table 6).

**TABLE 6:** Weibull distribution of the ESL-related AEs.

Cases n	Time to onset (days)		Weibull distribution				Failure type
			Scale parameter		Shape parameter		
	Media (IQR)	Min-MMin-Maxax	α 95% CI		β 95% CI		
109	27 (8–62)	1–803	53.29	37.88–68.70	0.69	0.59–0.78	Early failure

IQR: interquartile range.

## Discussion

Epilepsy is a common chronic brain disorder characterised by a long-standing tendency to recurrent seizures (Scheffer et al., 2017). Given that epilepsy is not a singular disease entity, it is crucial to be as precise as possible in the diagnosis, following the classification system of the International League Against Epilepsy (ILAE) (Fisher et al., 2017). Pathologically, both focal onset seizures and generalized onset seizures share the same mechanism of an imbalance between excitatory and inhibitory activity within a neuronal network (Pitkänen and Engel, 2014). Nevertheless, the spectrum of affected areas of the brain and the choice of anti-seizure medications represent two key differences between the two conditions (Nevitt et al., 2022). In clinical practice, the most common type of seizure is the partial-onset (focal) seizure, which is characterised by an initial activation of only part of one cerebral hemisphere (Galiana et al., 2017). Carbamazepine (CBZ), discovered serendipitously by Walter Schindler in 1953, has been the therapy of choice for this condition since the 1960s (Asadi-Pooya et al., 2023). As the inaugural member of the dibenzazepine carboxamide class, carbamazepine exerts its anti-seizure effects by blocking the voltage-gated sodium channel (VGSC), thereby reducing membrane excitability. However, it has still been limited by the occurrence of AEs and the complexity of its pharmacokinetics (Verrotti et al., 2014). In response to the growing demand for more effective anti-epileptic drugs, the second-generation (oxcarbazepine, OXC) and the third-generation (eslicarbazepine acetate, ESL) members of the dibenzazepine carboxamide family were developed. So far, ESL has shown promising results and demonstrated superiority over CBZ/OXC as an effective and safe alternative for treating partial-onset seizures (Rocamora et al., 2020). Its structurally configuration by a 5-carboxamide substitute at the 10,11 position of the dibenzazepine nucleus, leading to differences from CBZ/OXC in pharmacodynamics, pharmacokinetics, and metabolism (Shorvon et al., 2017). Different from traditional VGSC blockers interfering with the fast inactivation pathway, ESL’s active form (eslicarbazepine) enhances slow activation of VGSC and can also block the Cav3.2 T-type Ca2+ channel (Shorvon et al., 2017; Doeser et al., 2015). Additionally, unlike CBZ, ESL is not metabolized into the CBZ 10,11-epoxide, an active and potentially toxic compound, thus minimizing the enzymatic induction of the cytochrome P450 (CYP) system and autoinduction. In contrast to OXC, ESL is metabolized almost exclusively to the (S)-enantiomer with less than a 5% chiral conversion to the (R)-enantiomer, whereas OXC is converted to both (S)- and (R)- enantiomers in about a 4-5:1 proportion (Hwang et al., 2022). ESL’s stereoselective metabolism avoids the early peak in OXC concentration observed in plasma and CSF following immediate-release OXC administration, which correlates with OXC-related adverse events (dizziness, headache, etc.) (Keating, 2014). To date, several clinical studies, in addition to a descriptive analysis on the Argus Safety™ database, have provided evidence on the tolerability and safety profile of ESL (Guedes et al., 2023; Zhu et al., 2020). Building on the aforementioned foundation, this pharmacovigilance study further investigates ELS-related adverse events and their differences among subgroups using the FAERS database for the first time.

The FAERS database supports the FDA’s post-marketing monitoring of drugs and therapeutic biological products. It contains information on AEs and medication errors collected by the FDA, which can be analyzed by researchers to identify potential risk factors, high-risk groups, and emerging clinical safety issues. The data of the present study was retrospectively obtained from the FAERS database, beginning with the approval of ESL and extending to the most recent release in the first quarter of 2024. According to our findings, females (41.6%) accounted for a higher proportion of ESL-related ADRs compared to males (28.6%). It is notable that previous studies did not identify a significant difference in efficacy or AEs between the sexes. One potential explanation for this discrepancy may be the limited number of clinical study populations compared to the FAERS database, which may have resulted in undetected differences. In addition, a study by Amílcar Falcão suggested that women absorb more drugs than men, but it did not definitively confirm that gender influences the pharmacokinetics of ESL (Falcão et al., 2007). Nevertheless, this finding warrants further attention as a potential cause of increased AEs in women. Due to the spontaneous nature of the FAERS reporting system, the majority of our target cases lack information on age and weight. But it is also noteworthy that the remaining cases involved individuals aged 16–64.9 years old (24.6%) and weighing between 50-100 kg (9.1%), which exhibited the highest proportion of AEs. ESL is biotransformed into eslicarbazepine by hepatic hydrolysis and eliminated by renal excretion (Almeida et al., 2008). The pharmacokinetics of eslicarbazepine are linear, with the main pharmacokinetics parameters (C_max_ and AUC_0-24_) demonstrating dose-proportionality across the dose range of 400–2,400 mg/day (Elger et al., 2013). And its pharmacokinetics are not significantly affected by concomitant intake of food (Almeida et al., 2010) or age (Almeida et al., 2005). The discrepancy in AEs across weight categories suggests that the dosage applications may not be optimal. While among different age groups, the variation in the distribution of AEs may be attributed to the bimodal pattern of epilepsy, which shows peaks in infants under 1 year old and individuals over 50 years old (Thijs et al., 2019). Nevertheless, for example, a higher incidence of hyponatremia was observed, particularly in elderly patients who had experienced a stroke and had seizures (Gupta et al., 2015). This suggests that specific PTs may have differential occurrences across age groups, which require further investigation. The most common AEs associated with ELS are dizziness, headache, fatigue, and diplopia, which are typically of mild or moderate severity (Ley et al., 2015). It is also possible that serious dermatological and electrolyte AEs may be caused by ESL (Guedes et al., 2023). However, their incidence is relatively rare, consistent with our findings that serious adverse outcomes such as death and life-threatening conditions accounted for a small fraction of ESL-related outcomes (4.1%) (Shorvon et al., 2017; Zhu et al., 2020). With regard to the countries from which the reports originated, the majority were from the United States and Portugal. As China has not yet established a definitive timeline for ESL, it is not possible to identify any relevant adverse reaction data, nor to analyse the racial differences from other countries. In Korea, a randomized controlled trial (RCT) conducted by Hwang involving 29 Korean and 20 White subjects suggests that ESL was well-tolerated in healthy Korean and White subjects, and that its pharmacokinetic (PK) characteristics were comparable between the two ethnic groups (Hwang et al., 2022). However, a series of studies on ESL pharmacogenetics have demonstrated a significant association between drug resistance and the increased efflux of eslicarbazepine by P-glycoprotein (Pgp, ABCB1, or MDR1) *in vitro* (Zhang et al., 2011). Similarly, *in vivo* evidence indicated that one of the three ABCB1 common polymorphisms, ABCB1 C1236 T C/C diplotype, has been identified as a significant risk factor for the occurrence of AEs (Zubiaur et al., 2021). The divergent conclusions underscore the necessity for further studies to be conducted in order to facilitate a comparison of the results obtained here. We also observed that the number of AEs reports of ESL exhibited an upward trend prior to 2019, followed by a downward trend afterwards. The global sales volume of ESL is exhibiting a growth rate of approximately 10% per annum, whereas the increase in the use of ESL has not resulted in a continuous increase in the number of AE reports. We postulated that this may be attributable to the more judicious application of ESL, guided by medical professionals, although it is necessary to monitor whether the downward trend in adverse reactions to ESL will persist in the future.

At the system-organism level, ADRs related to ESL (n = 5,719) were distributed across 27 SOCs, with the nervous system being the most affected (n = 1,676). Further disproportionality analysis identified five significant SOCs that exceed at least one of the four algorithms’ threshold. “Nervous system disorders” was both the positive and most frequent affected SOC related to ESL, which aligns with the drug label’s mention of dizziness, somnolence, disturbance in gait and coordination, cognitive dysfunction, and visual change as the most frequent AEs. Although the incidence of these AEs is relatively high, the intensity is often mild or moderate (Ley et al., 2015; Sperling et al., 2015a). Another SOC commonly associated with anti-epileptic drugs (AED) is psychiatric disorders, which is also identified as a positive signal for ESL in this study. In some perspectives, psychiatric conditions are frequently observed in individuals diagnosed with epilepsy, while AEDs such as ESL may potentially elevate the risk of psychiatric AEs, including depression and suicidal ideation (Andermann et al., 2018). On the contrary, a recent study, which employed post-hoc analysis of data from three Phase III RCTs, found no discernible difference in the incidence of psychiatric AEs between patients who received a placebo and those who received ESL (Altalib et al., 2022). Based on our research, depression and suicidal ideation ranked prominently within the SOC of “Psychiatric disorders” and yielded positive signals. Although our findings do not indicate that ESL increases the risk of suicide, they do suggest that patients with epilepsy who are taking ESL may be at risk of suicide. Consequently, we also recommend that continuous attention be paid to the emergence or worsening of depressive symptoms, as well as any unusual changes in mood or behaviour, or the emergence of suicidal thoughts, behaviour or thoughts about self-harm during the administration of ESL medication. Such occurrences should be promptly reported to the relevant health providers, as the consequences may be fatal. Signal of “Metabolism and nutrition disorders” was positive at SOC level, primarily due to the ESL-related AEs of hyponatremia, which was also an important AE for CBZ and OXC. Compared to other AED drugs, ESL appears to have a lower incidence of hyponatremia, occurring in only 0.6%–1.5% of patients. The reduction in sodium levels was most pronounced within 8 weeks of treatment, after which the levels remained relatively stable. Results from a Phase III clinical trial showed that 1.4% of patients receiving a dosage of 1,200 mg ESL experienced severe hyponatremia leading to discontinuation, while none of the patients taking 800 mg experienced this AE prompting withdrawal (Sperling et al., 2015b). Monitoring serum sodium levels is necessary for patients (particularly the elderly), who are undergoing maintenance treatment with ESL. This is especially important if the patient is taking other medications (e.g., diuretic, vinca alkaloids, platinum, SSRIs, TCAs, PPIs, CCBs) known to lower serum sodium levels. Additionally, when symptoms of hyponatremia such as nausea, vomiting, malaise, or headache develop, serum sodium level monitoring should be carried out promptly. “Injury, poisoning, and procedural complications” exhibited a positive signal at SOC level mainly attributed to issues related to dosage. As with other AEDs, ESL should be introduced gradually and doses increased in steps, contingent on the presenting symptoms. The drug is recommended at a dosage of 400 mg once daily, with weekly titration to the maximum tolerated dose (800–1,600 mg once daily) if seizures persist. Hence, the process of adjusting drug doses is relatively complex and vulnerable to unsuitableness, whether during the initial treatment or the maintenance phase of ESL. In the event of any intolerability issues or a lack of efficacy at the maximum tolerated dose being observed, a reduction in dose or an alteration in the first-line drug should be considered. This, in turn, also generates a positive signal at the SOC level in the context of “Surgical and medical procedures” regarding the interruption or cessation of therapy. Notably, “brain operation” was a positive PT under this SOC, however, it is considered to be correlated with the progression of the underlying medical condition rather than being AEs caused by ESL itself.

At the PT level, a total of 86 effective PTs were identified as meeting the criteria of all four algorithms simultaneously. Among these PTs, suicidal ideation, Stevens-Johnson syndrome, drug reaction with eosinophilia and systemic symptoms (DRESS), toxic skin eruption, dermatitis exfoliative, pharyngeal oedema, hyponatremia, somnolence, balance disorder, memory impairment, amnesia, diplopia, eye movement disorder, and ammonia increased were in accordance with the drug instructions. Furthermore, the study identified a number of additional AEs, including seizure/epilepsy (different presentations), over/under dose, agitation, anger, stress, aggression, fear, irritability, increased appetite, change of bowel habit, and so forth, which were not listed in the instructions. Here, it is noticeable that several unexpected PTs belonging to psychiatric disorders were detected. Apart from the SOC, PTs could also be categorized at the SMQ level for their similar clinical presentation or shared pathological pathway. At the SMQ level, the top five classification in number of cases for the 86 significant PTs were as follows: convulsions, hyponatremia/SIADH, medication errors, hypersensitivity, and depression and suicide/self-injury. The reason why convulsions was the most common may be partially attributed to the inherent drug-resistant nature of epilepsy, which could be observed in approximately one-third of cases. Along with the fact that even ESL showed its short-term effectiveness with proportion of responder rates (patients with ≥50% reduction in standardized seizure frequency) ranged from 33.8% to 43.1% in a pooled analysis of four RCTs (Elger et al., 2017), as well as one-year long-term effectiveness with a responder rate of 60.3% and seizure freedom rate of 14.7% (Lee et al., 2024), the remaining individuals may still report seizure/epilepsy to FAERS during the initial or maintenance treatment period. To assess the serious and specific safety events associated with ESL administration, we further examined the DME list to identify valuable positive signals based on these 86 PTs. Three signals including dermatitis exfoliative, stevens-johnson syndrome (SJS), and drug reaction with eosinophilia and systemic symptoms (DRESS) were consistent with the PT signals on the DME list, focusing on the SOC of “skin and subcutaneous tissue disorders.” Two additional PTs of toxic skin eruption and rash maculo-papular were also found under the corresponding SOC, yet they were seen as different stages of the same dermatological condition spectrum. The pathogenesis may be attributed to the damage caused to endothelial cells by 10,11-epoxide metabolites, which in turn led to the release of cellular antigens and the occurrence of epitope spreading, resulting in an autoimmune response. However, further clinical confirmation is needed to determine the effect of ESL on the immune system. Due to the strong association between life-threatening cutaneous AEs and the HLA-B*15:02 or HLA-A*31:01 allele, it may be advisable to consider screening for human leucocyte antigen (HLA) before initiating ESL in individuals of Asian descent (Amstutz et al., 2014). A report has documented the successful alteration of ESL without encountering any AEs in a patient with the HLA-A*31:01 haplotype, who previously experienced a severe cutaneous reaction following administration of CBZ (Kay et al., 2017). Nevertheless, it was proposed that ESL should be considered with great caution in the context of the HLA-B*15:02 or HLA-A*31:01 haplotype, where the potential benefits may not outweigh the risks. The instructions for ESL explicitly delineate the AEs associated with serious dermatologic reactions, including SJS and toxic epidermal necrolysis (TEN), and DRESS, which may be fatal and necessitate heightened caution. Although serious AEs occur infrequently during treatment with ESL, it is important to consider discontinuing ESL immediately if any dermatologic reactions or early signs of hypersensitivity appear (Galiana et al., 2017). Patients and caregivers should also be adequately informed and educated about potential indicators associated with serious AEs.

Subgroup analysis provides a novel perspective on the AEs of ESL in different gender and age groups. Previous research indicates that while hyponatremia is more prevalent in the elderly population, there is no significant variance in ESL-related AEs across different gender and age groups (Falcão et al., 2007; Almeida et al., 2005; Elger et al., 2017). However, our findings indicate that males are more commonly affected by seizures, agitation, and aggression than females. Compared to the adult group, seizures, hyponatremia, and confusional states were more frequently observed in the elderly population, while aggression, irritability, DRESS, and abnormal behavior were found to be more common in the pediatric population. Furthermore, both the paediatric and geriatric individuals exhibited a higher proportion of agitation than the adult group. Consequently, these findings indicate the necessity to monitor the occurrence of AEs in specific population subgroups, with particular attention being paid to DRESS in children. Nevertheless, it is important to recognize that these novel perspectives are merely indicative evidence, and that further clinical studies are required in order to substantiate these conclusions.

The TTO analysis revealed a median onset time of 27 days, with the majority of AEs occurring within the initial 30 days following exposure to ESL. Furthermore, 79.81% of all AEs occurred during the first 3 months. This finding was consistent with the results of a one-year open-label extension study, which indicated that ESL-related AEs were most prevalent during the initial 3 months of treatment (Halász et al., 2010). Consequently, it was important that clinicians maintain close contact with patients who were utilizing ESL, particularly within the initial 90-day period. Notable, AEs may still occur up to a year later, although in a reduced proportion. The Weibull distribution tests indicated the presence of an early failure type, which suggests a decline in the occurrence of AEs over time.

## Limitations

The limitations of this study can be attributed to the following factors: Firstly, it should be noted that the FAERS database is a spontaneous reporting database, and as such, the quality and quantity of information provided by reporters is not subject to rigorous control. Other general limitations of pharmacovigilance, such as under-reporting, difficulty in identifying low risks, and the difficulty or impracticality of quantifying risks, are also inevitable in this study. Secondly, while the utilization of analytical methodologies can undoubtedly facilitate our comprehension of the strength of the relationship between drugs and AEs, it is imperative to recognize that well-designed clinical trials remain indispensable in determining causation. Thirdly, certain confounding factors, such as potential interactions between medications, pre-existing medical conditions, and the use of multiple drugs, were not accounted for in the study. Further investigation is still required through the implementation of extensive clinical studies in order to address the aforementioned issues. Despite the limitations of the FAERS database for pharmacovigilance research, the comprehensive analysis AEs associated with ESL in this study provides substantial evidence for the safe usage of ESL and further clinical investigation.

## Conclusion

This comprehensive and systematic pharmacovigilance analysis demonstrates several common and rare side effects of ESL use and variations among different gender and age groups. Careful monitoring of dermatitis exfoliative, stevens-johnson syndrome is recommended, particularly DRESS in children and hyponatremia in the elderly. The current data supports the known safety and tolerability profile of ESL, with most AEs being non-serious. As such, it is our conviction that the investigation of novel indications and the implementation of more prudent clinical applications will result in substantial benefits for a broader range of patients.

## Data Availability

The original contributions presented in the study are included in the article/Supplementary Material, further inquiries can be directed to the corresponding authors.
