# Supplementary material for: A real-world pharmacovigilance analysis of eslicarbazepine acetate using the FDA adverse events reporting system (FAERS) database from 2013 (Q4) to 2024 (Q1)
Source: Front Pharmacol. 2024 Sep 20;15:1463560. doi: 10.3389/fphar.2024.1463560 (PMC11449726; doi:10.3389/fphar.2024.1463560)
Supplement: Supplementary file 2 [file Table3.docx]

| **Supplementary table 3. Logistic regression of different age groups** | | | | | | | | |
| --- | --- | --- | --- | --- | --- | --- | --- | --- |
|  | **Children (aged < 18 years)** | | | | **Elderly (aged > 65 years)** | | | |
| **PT** | **Crude**  **OR(95% CI)** | **P value** | **Adjusted OR**  **(95% CI)** | **P value** | **Crude**  **OR(95% CI)** | **P value** | **Adjusted**  **OR(95% CI)** | **P value** |
| **Seizure** | 1.31 (0.79-2.15) | 0.286 | 1.30 (0.79-2.14) | 0.301 | 0.64 (0.40-0.99) | **0.049^*^** | 0.63 (0.40-1.00) | 0.051 |
| **Hyponatremia** | 0.72 (0.21-2.45) | 0.604 | 0.73 (0.21-2.48) | 0.615 | 2.61 (1.33-5.11) | **0.005^*^** | 2.60 (1.32-5.09) | **0.005^*^** |
| Somnolence | 0.95 (0.27-3.29) | 0.940 | 0.95 (0.27-3.31) | 0.946 | 1.04 (0.38-2.83) | 0.939 | 1.03 (0.38-2.82) | 0.944 |
| Therapy interrupted | 0.52 (0.06-4.09) | 0.538 | 0.52 (0.06-4.09) | 0.538 | 0.68 (0.15-3.11) | 0.626 | 0.68 (0.15-3.11) | 0.626 |
| Memory impairment | 0.39 (0.05-2.98) | 0.364 | 0.38 (0.05-2.97) | 0.363 | 1.31 (0.47-3.65) | 0.605 | 1.31 (0.47-3.66) | 0.603 |
| **Confusional state** | 0.00 (0.00-1.1e+11) | 0.984 | 0.00 (0.00-3.3e+14) | 0.990 | 2.87 (1.30-6.31) | **0.009^*^** | 2.85 (1.29-6.29) | **0.009^*^** |
| Suicidal ideation | 1.62 (0.52-5.00) | 0.396 | 1.62 (0.52-4.99) | 0.400 | 0.25 (0.03-1.92) | 0.184 | 0.25 (0.03-1.93) | 0.185 |
| Drug dose titration not performed | 6.44 (0.39-104.18) | 0.189 | 6.37 (0.39-103.16) | 0.192 | 0.00 (0-7.2e+198) | 0.996 | 0.00 (0-2.6e+198) | 0.996 |
| Generalized tonic-clonic seizure | 0.32 (0.04-2.47) | 0.278 | 0.33 (0.04-2.49) | 0.282 | 0.21 (0.02-1.59) | 0.132 | 0.21 (0.02-1.58) | 0.131 |
| Blood sodium decreased | 0.57 (0.07-4.50) | 0.597 | 0.59 (0.07-4.72) | 0.625 | 1.53 (0.47-4.89) | 0.475 | 1.49 (0.46-4.83) | 0.500 |
| Epilepsy | 1.06 (0.12-8.95) | 0.956 | 1.05 (0.12-8.92) | 0.958 | 0.00 (0-8.5e+62) | 0.992 | 0.00 (0-8.2e+62) | 0.992 |
| Partial seizures | 2.44 (0.63-9.44) | 0.194 | 2.47 (0.64-9.54) | 0.190 | 0.51 (0.06-4.15) | 0.533 | 0.51 (0.06-4.13) | 0.530 |
| Underdose | 0.00 (0-1.9e+33) | 0.990 | 0.00 (0-8.9e+32) | 0.990 | 1.87 (0.56-6.20) | 0.302 | 1.85 (0.56-6.15) | 0.312 |
| Balance disorder | 0.00 (0-2.3e+36) | 0.990 | 0.00 (0-2.0e+36) | 0.990 | 2.42 (0.69-8.42) | 0.164 | 2.43 (0.70-8.48) | 0.161 |
| Prescribed underdose | 1.27 (0.14-11.09) | 0.824 | 1.26 (0.14-10.94) | 0.834 | 3.40 (0.90-12.90) | 0.071 | 3.44 (0.90-13.05) | 0.069 |
| Therapy cessation | 1.42 (0.30-6.74) | 0.653 | 1.45 (0.30-6.89) | 0.635 | 0.45 (0.05-3.64) | 0.459 | 0.45 (0.05-3.59) | 0.452 |
| **Agitation** | 4.94 (1.08-22.54) | **0.039^*^** | 4.80 (1.04-22.11) | **0.044^*^** | 4.27 (1.05-17.33) | **0.042^*^** | 4.45 (1.09-18.21) | **0.038^*^** |
| Stress | 0.00 (0.00-3.7e+54) | 0.993 | 0.00 (0.00-5.6e+53) | 0.993 | 0.00 (0.00-4.1e+42) | 0.992 | 0.00 (0.00-9.7e+41) | 0.992 |
| Petit mal epilepsy | 0.00 (0.00-1.3e+86) | 0.994 | 0.00 (0.00-9.2e+85) | 0.994 | 0.83 (0.09-7.16) | 0.865 | 0.82 (0.09-7.13) | 0.861 |
| Brain operation | 0.00 (0.00-1.2e+38) | 0.990 | 0.00 (0.00-9.8e+37) | 0.990 | 0.45 (0.05-3.64) | 0.459 | 0.45 (0.05-3.61) | 0.455 |
| Amnesia | 0.00 (0.00-5.8e+79) | 0.994 | 0.00 (0.00-5.3e+79) | 0.994 | 1.67 (0.32-8.73) | 0.542 | 1.67 (0.32-8.76) | 0.539 |
| **Aggression** | 19.88 (2.04-193.85) | **0.010^*^** | 19.66 (2.01-191.84) | **0.010^*^** | 8.43 (0.75-193.87) | 0.083 | 8.50 (0.76-194.73) | 0.082 |
| **Irritability** | 4.43 (1.22-16.11) | **0.024^*^** | 4.56 (1.25-16.64) | **0.022^*^** | 2.10 (0.51-8.55) | 0.298 | 2.08 (0.51-8.47) | 0.306 |
| Prescribed overdose | 0.00 (0.00-1.1e+135) | 0.996 | 0.00 (0.00-6.5e+133) | 0.996 | 0.00 (0.00-3.8e+107) | 0.995 | 0.00 (0.00-3.9e+106) | 0.995 |
| Speech disorder | 0.00 (0.00-1.7e+38) | 0.997 | 0.00 (0.00-5.0e+37) | 0.997 | 2.83 (0.78-10.21) | 0.111 | 2.80 (0.77-10.12) | 0.116 |
| **Drug reaction with eosinophilia and systemic symptoms** | 9.92 (1.63-60.42) | **0.013^*^** | 9.93 (1.63-60.58) | **0.013^*^** | 0.00 (0.00-1.8e+74) | 0.993 | 0.00 (0.00-1.8e+74) | 0.993 |
| Labelled drug-drug interaction medication error | 4.33 (0.71-26.41) | 0.111 | 4.38 (0.71-26.69) | 0.109 | 1.38 (0.14-13.46) | 0.778 | 1.38 (0.14-13.39) | 0.781 |
| Disturbance in attention | 0.00 (0.00-1.7e+94) | 0.994 | 0.00 (0.00-1.0e+93) | 0.994 | 1.03 (0.11-9.38) | 0.973 | 1.02 (0.11-9.25) | 0.984 |
| **Abnormal behaviour** | 26.89 (2.96-244.18) | **0.003^*^** | 26.63 (2.93-241.93) | **0.004^*^** | 4.18 (0.26-67.35) | 0.313 | 4.21 (0.26-67.88) | 0.311 |
| Treatment noncompliance | 1.60 (0.17-14.52) | 0.676 | 1.60 (0.17-14.53) | 0.676 | 0.00 (0.00-4.4e+74) | 0.992 | 0.00 (0.00-4.4e+74) | 0.992 |
| Anger | 2.14 (0.22-20.84) | 0.513 | 2.13 (0.22-20.84) | 0.513 | 1.38 (0.14-13.46) | 0.77 | 1.38 (0.14-13.47) | 0.77 |
| Decreased activity | 0.00 (0.00-7.6e+42) | 0.990 | 0.00 (0.00-3.6e+42) | 0.990 | 0.59 (0.07-4.84) | 0.623 | 0.58 (0.07-4.79) | 0.615 |
| Fear | 0.00 (0.00-1.7e+94) | 0.994 | 0.00 (0.00-1.0e+93) | 0.994 | 1.03 (0.11-9.38) | 0.973 | 1.02 (0.11-9.25) | 0.984 |
| Hypersomnia | 6.52 (0.90-47.03) | 0.063 | 6.44 (0.89-46.50) | 0.065 | 4.21 (0.58-30.21) | 0.153 | 4.24 (0.59-30.50) | 0.151 |
| Ataxia | 0.00 (0.00-1.7e+94) | 0.994 | 0.00 (0.00-1.6e+94) | 0.994 | 1.03 (0.11-9.38) | 0.973 | 1.04 (0.11-9.39) | 0.973 |
| Restlessness | 1.60 (0.17-14.52) | 0.676 | 1.64 (0.18-14.97) | 0.66 | 0.00 (0.00-4.4e+74) | 0.992 | 0.00 (0.00-4.4e+73) | 0.992 |
| Inappropriate antidiuretic hormone secretion | 0.00 (0.00-1.7e+94) | 0.994 | 0.00 (0.00-1.0e+93) | 0.994 | 1.03 (0.11-9.38) | 0.973 | 1.05 (0.11-9.55) | 0.961 |
| Therapeutic product effect increased | 3.21 (0.28-35.91) | 0.343 | 3.41 (0.30-38.50) | 0.320 | 2.08 (0.18-23.20) | 0.550 | 2.03 (0.18-22.70) | 0.556 |
| Eosinophilia | 0.00 (0.00-3.7e+121) | 0.994 | 0.00 (0.00-2.8e+121) | 0.994 | 2.08 (0.18-23.20) | 0.550 | 2.07 (0.18-23.12) | 0.552 |
| Mental impairment | 0.00 (0.00-1.1e+86) | 0.994 | 0.00 (0.00-2.6e+84) | 0.994 | 0.83 (0.09-7.16) | 0.865 | 0.81 (0.09-7.05) | 0.852 |
| Bradyphrenia | 0.00 (0.00-5.8e+248) | 0.996 | 0.00 (0.00-6.8e+247) | 0.996 | 4.18 (0.25-67.34) | 0.313 | 4.22 (0.26-68.06) | 0.310 |
| Stevens-Johnson syndrome | 6.44 (0.39-104.18) | 0.189 | 6.50 (0.40-105.21) | 0.188 | 4.18 (0.26-67.35) | 0.313 | 4.16 (0.25-67.04) | 0.315 |
| Intentional underdose | 1.60 (0.17-14.52) | 0.676 | 1.69 (0.18-15.54) | 0.641 | 0.00 (0.00-4.4e+74) | 0.992 | 0.00 (0.00-8.4e+197) | 0.997 |
| Psychomotor hyperactivity | 13.07 (1.17-146.02) | 0.037 | 13.22 (1.18-147.92) | 0.036 | 0.00 (0.00-7.8e+161) | 0.996 | 0.00 (0.00-3.9e+161) | 0.996 |
| Trigeminal neuralgia | 0.00 (0.00-5.8e+248) | 0.996 | 0.00 (0.00-4.4e+247) | 0.996 | 4.18 (0.26-67.35) | 0.313 | 4.07 (0.25-65.92) | 0.323 |
| Slow speech | 0.00 (0.00-5.8e+248) | 0.996 | 0.00 (0.00-5.8e+248) | 0.996 | 4.18 (0.26-67.35) | 0.313 | 4.07 (0.25-65.92) | 0.323 |
| Epileptic aura | 0.00 (0.00-5.8e+248) | 0.996 | 0.00 (0.00-6.8e+247) | 0.996 | 4.18 (0.26-67.35) | 0.313 | 4.22 (0.26,68.06) | 0.310 |
| Staring | 0.00 (0.00-5.8e+248) | 0.996 | 0.00 (0.00-5.8e+248) | 0.996 | 4.18 (0.26-67.35) | 0.313 | 4.07 (0.25-65.92) | 0.323 |
| Communication disorder | 0.00 (0.00-3.7e+121) | 0.994 | 0.00 (0.00-2.8e+121) | 0.994 | 2.08 (0.18-23.20) | 0.550 | 2.07 (0.18-23.12) | 0.552 |

The adult group was the reference group. The PTs without OR value were exclude from the table. PT: Preferred term, OR: odds ratio. * P<0.05
